# Survey data on strategic alignment in multispecialty hospitals: Implementing a balanced scorecard approach for optimal performance

**DOI:** 10.1016/j.dib.2025.111329

**Published:** 2025-01-31

**Authors:** Shefali Mohan, Rohit Kushwaha, M. Venkatesan, Ramya Singh, Manish Mishra

**Affiliations:** a Amity Business School, Amity University, Malhaur, Gomti Nagar, Lucknow, India; b Indian Institute of Foreign Trade, New Delhi, India

**Keywords:** Strategic alignment, Multispecialty hospitals, Balanced scorecard, Optimal performance, Healthcare management, Performance management

## Abstract

This dataset investigates the relationship between Strategic Alignment, Balanced Scorecard (BSC), and Optimal Performance (OP) in multispecialty hospitals. Data were collected using a cross-sectional design from 530 healthcare professionals in four cities in India: Lucknow, Varanasi, Kanpur, and Prayagraj. The data collection process involved Interviews and administering structured questionnaires to participants, capturing their perceptions and experiences related to strategic alignment, BSC implementation, and performance outcomes.

The dataset comprises responses from a diverse group of healthcare professionals, including doctors, nurses, administrators, and support staff. Variables in the dataset include demographic information, measures of strategic alignment, BSC implementation metrics, and performance indicators. Sentiment analysis and Descriptive statistics were used to analyze the data, Correlation and regression analyses were employed to explore the relationships between the constructs.

This dataset offers significant reuse potential for researchers interested in examining strategic management practices in healthcare settings. It provides a comprehensive snapshot of how strategic alignment and performance measurement frameworks are perceived and implemented across multiple hospitals in different urban regions. The dataset can be leveraged for comparative studies, validation of performance measurement tools, and further exploration of factors influencing optimal performance in healthcare organizations.

Specifications Table

**Table:** 

Subject	Human Resource
Specific subject area	Leadership
Data format	Raw, Analyzed, Filtered
Data collection	The data were collected using Interview and a structured questionnaire distributed in person. The questionnaire, derived from existing Balance Score Card and Optimal Performance literature, covered demographics, BSC perspectives, and OP metrics. Respondents were purposively sampled from multispecialty hospitals, targeting Healthcare Professional (Doctor, Nurse, Technician, etc.), Administrative, Management Staff, Support Staff (Clerical, Maintenance, etc.). Data were analyzed using NVivo 14 and SPSS (IBM SPSS Statistics 26).
Data source location	The data were collected from multispecialty hospitals located in Lucknow, Varanasi, Kanpur, and Prayagraj, India.
Data accessibility	Repository name: Mendeley Data: Mendeley Data. Data identification number: 10.17632/s46hxyhzwx.1 Direct URL to data: https://data.mendeley.com/datasets/s46hxyhzwx/1
Related research article	Bahadur, S., Kushwaha, R., Venkatesan, M., Singh, R., & Mishra, M. (2024). Strategic alignment in multispecialty hospitals: Implementing a balanced scorecard approach for optimal performance. The Scientific Temper, 15(01), 1915–1923 [1].

## 1 Value of the Data

• This data offers a detailed analysis of the application of the Balanced Scorecard method in aligning hospital strategies. It is a crucial resource for hospital administrators and policymakers, providing insights necessary for optimizing hospital performance and enhancing decision-making processes.
• The dataset serves as a valuable tool for researchers aiming to investigate the variations in Balanced Scorecard implementation across different hospitals. By examining the specific successes and challenges documented, researchers can refine and adapt these practices for various healthcare environments.
• The data includes quantitative metrics related to patient care quality, operational efficiency, and financial sustainability. These metrics are essential for assessing hospital performance and developing benchmarks for evaluating the impact of strategic alignment initiatives in healthcare institutions.
• This dataset allows researchers to delve into the factors influencing the effectiveness of the Balanced Scorecard in different hospital settings. A deeper understanding of these factors can lead to the development of tailored strategies that support effective strategic alignment and improve overall hospital services.

## 2 Background

The motivation behind compiling this dataset was driven by the need to enhance strategic alignment and performance in multispecialty hospitals. The healthcare sector, especially multispecialty hospitals, operates in a complex environment where various medical disciplines intersect. Achieving strategic alignment in these settings involves aligning organizational goals, resources, and activities across different specialties to ensure cohesive and synergistic healthcare delivery.

The theoretical background of this research is rooted in the Balanced Scorecard (BSC) framework, introduced by Kaplan and Norton in the early 1990s [2]. The BSC is a strategic management tool designed to translate an organization's vision and strategy into tangible objectives and performance indicators. By integrating performance measures across different perspectives, such as financial management, patient satisfaction, operational efficiency, and employee engagement, hospitals can effectively navigate the complexities of the healthcare landscape while focusing on delivering high- quality care and operational excellence.

This data article complements the original research article by providing empirical data on the implementation of the Balanced Scorecard approach in multispecialty hospitals. It highlights the relationship between strategic alignment, BSC implementation, and organizational performance (OP), offering insights into the challenges and benefits of adopting this strategic management tool in the healthcare context.

## 3 Data Description

The dataset comprises responses from a survey conducted among professionals in multispecialty hospitals across four cities: Lucknow, Varanasi, Kanpur, and Prayagraj. Data were collected from 530 [15] healthcare professionals across four cities in India. The survey aimed to gather insights on Strategic Alignment, Balanced Scorecard Approach, and Optimal Performance of these hospitals. The dataset includes demographic characteristics and responses to the main sections of the study.

Table 1 outlines the demographic characteristics of the respondents, focusing on key variables such as occupation, years of experience, age, and gender. These variables provide a detailed snapshot of the sample's professional and personal background.

**Table 1:** Demographic profile.

S No.	Demographic Characteristics	Category	N	%
1	Gender	Male	312	58.9
		Female	218	41.1
2	Age group	18-28 Years	112	21.1
		29-36 Years	142	26.8
		37-44 Years	124	23.4
		Above 45 years	152	28.7
3	Occupation	Healthcare Professional (Doctor, Nurse, Technician, etc.)	203	38.4
		Administrative	145	27.4
		Management Staff	94	17.7
		Support Staff (Clerical, Maintenance, etc.)	88	16.6
44	Experience	1-5 years	163	30.8
		6 to 10 years	91	17.2
		11 to 15 years	135	25.5
		16 to 20 years	82	15.5
		More than 20 years	59	11.1

**Fig. 1:**
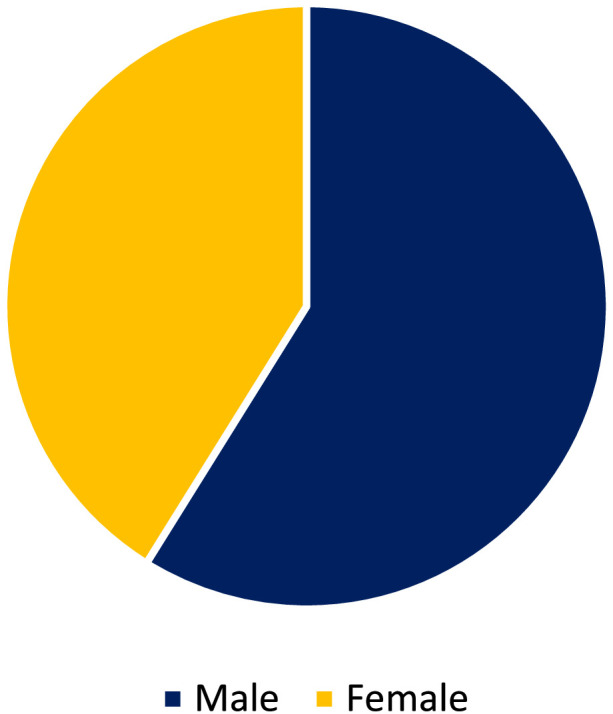
Gender distribution.

Fig. 1 reveals that the majority of respondents were male (59.0%), while females accounted for 41.0%.

**Fig. 2:**
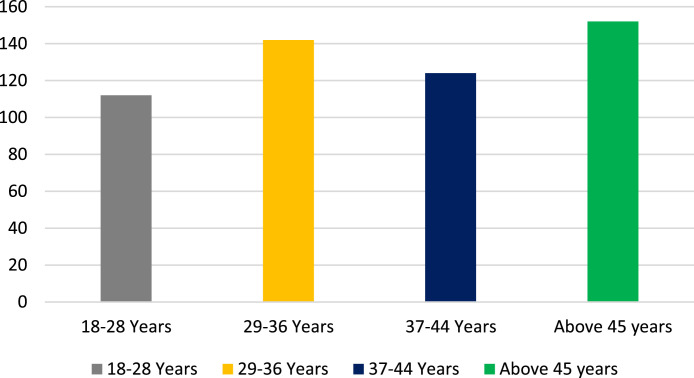
Age distribution.

Fig. 2 shows that Age distribution showed that the highest proportion of respondents fell into the “Above 45 years” category (37.6%), followed by “37-44 Years” (25.8%), “29-36 Years” (20.6%), and “18-28 Years” (16.0%).

**Fig. 3:**
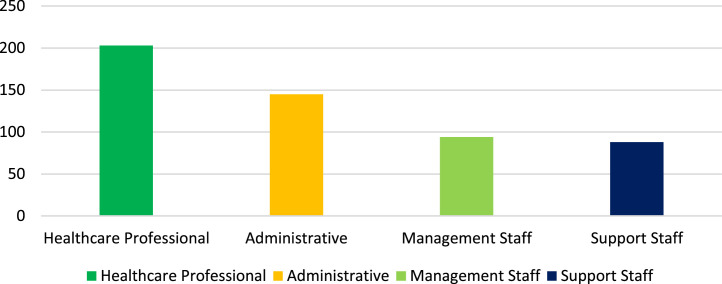
Occupation.

Fig. 3 displays that occupation, the respondents were categorized as healthcare professionals (38.0%), administrative staff (27.0%), management staff (17.5%), and support staff (16.4%).

**Fig. 4:**
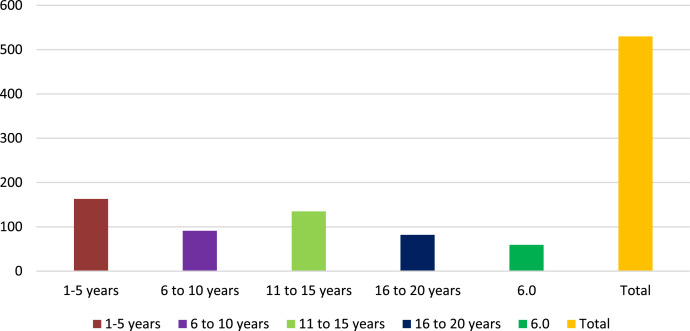
Year of experience.

Fig. 4 displays the years of experience in the healthcare sector, the highest percentage of respondents had 5 years or less of experience (23.7%), followed by those with 6 to 10 years (20.1%), 11 to 15 years (14.1%), 16 to 20 years (8.6%), and more than 20 years (6.2%).

Survey question was used 5-point Likert scale to measure responses related to Strategic Alignment, Balanced Scorecard Approach, and Optimal Performance of multispecialty hospitals, where 1 represented “Strongly Disagree” and 5 represented “Strongly Agree.” This structured approach provided a comprehensive understanding of the strategic and operational dynamics within these hospitals.

Codebooks raw (.xlsx), research questionnaire (.docx), scale development (.xlsx), SPSS file (.sav) and Codebook are available in https://data.mendeley.com/datasets/s46hxyhzwx/1

## 4 Experimental Design, Materials and Methods

In this research, we employed a mixed methods approach to ensure a comprehensive understanding of the healthcare sector from various stakeholders, including doctors, nurses, managers, and office staff. We gathered data through interviews, structured online surveys, and physical questionnaires. This diverse collection method allowed us to reach participants in multiple locations, providing both in-depth qualitative insights and broad quantitative data. The study was conducted across multispecialty hospitals in Lucknow, Varanasi, Kanpur, and Prayagraj, India, capturing a wide range of experiences and perspectives.

**Fig. 5:**
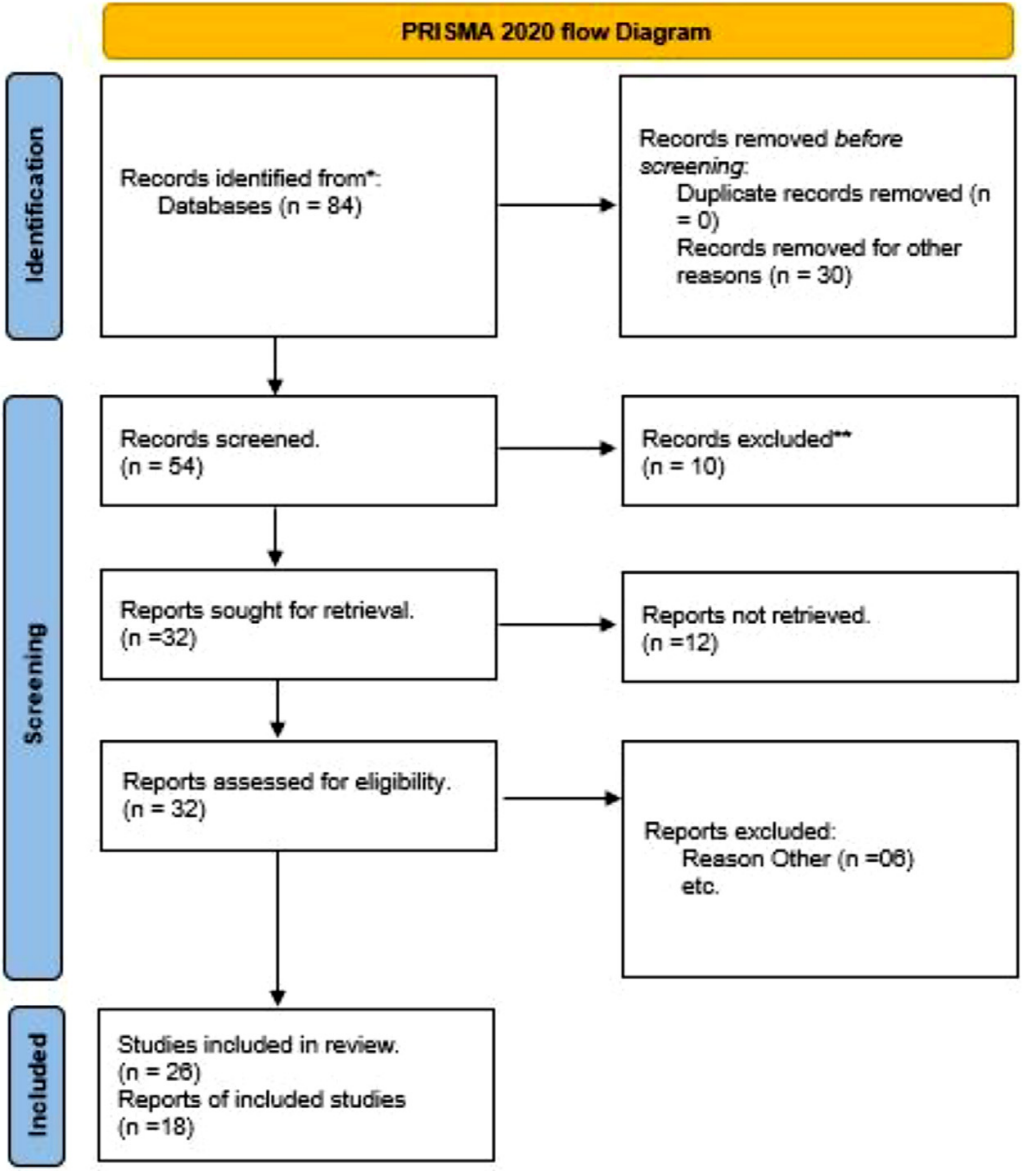
PRISMA flow chart.

To develop the structured questionnaire, a comprehensive literature review was conducted using the Scopus database, focusing on “Balanced Scorecard,” “strategic alignment,” and “organizational performance.” The search was restricted to final-stage, English-language articles within the Business and Management subject area, resulting in an initial set of 84 documents. After applying specific inclusion and exclusion criteria, this was refined to 18 relevant articles. Fig. 5 displays the PRISMA framework, which was used to guide the selection process, ensuring a systematic approach to the review. Insights from these searches informed the development of the questionnaire, ensuring it effectively addresses key aspects of the Balanced Scorecard, strategic alignment, and organizational performance.

Table 2, with items measured on a 5-point Likert scale ranging from “strongly disagree” to “strongly agree.” A purposive sampling technique was used to select 150 respondents, ensuring representation from different specialties and departments [3]. Data collection was conducted through online surveys and physical questionnaires distributed at the hospitals. Data were analyzed using IBM SPSS Statistics (version 26). Survey responses were coded and input into SPSS, and data normalization involved standardizing responses for consistency. Table 3 displays the descriptive statistics which includes means, standard deviations, and frequency distributions. The reliability of the survey instrument was assessed using Cronbach's alpha, with reliability assessments for the observed constructs showing acceptable levels of internal consistency.

**Table 2:** Construct, operational definition and items.

Construct	Operational Definition	Item	Source
Balanced Scorecard	The Balanced Scorecard is a strategic management framework comprising financial, customer, internal processes, and learning/growth perspectives. It helps organizations align goals, monitor performance, and make informed decisions by considering a balanced view of key performance indicators across these four dimensions.	I believe the Balanced Scorecard facilitate communication and alignment of strategic goals across different departments within the hospital. From a financial standpoint, Key performance indicators (KPIs) are considered crucial for ensuring optimal performance in multispecialty hospitals. The financial objectives outlined in the Balanced Scorecard align seamlessly with our hospital's broader financial goals, fostering strategic harmony and optimal financial performance. In my experience, Balanced Scorecard contributed to fostering a culture of continuous learning, innovation, and professional development among healthcare professionals in my hospital.	[4] [4] [5] [6]
		Financial objectives outlined in the Balanced Scorecard align with the broader financial goals of your hospital.	[4]
		I feel the Balanced Scorecard facilitate communication and alignment of strategic goals across different departments within the hospital.	[5]
		I believe the financial, customer, internal processes, and learning and growth perspectives integrated into the Balanced Scorecard to drive holistic performance assessment.	[4]
		In my opinion the Balanced Scorecard has influenced decision-making processes and resource allocation in your hospital to enhance overall performance.	[6]
Strategic Alignment	Strategic alignment refers to the harmonization of an organization's goals, activities, and resources with its overarching strategic objectives. It ensures that all aspects of the organization work cohesively towards a common vision, optimizing performance and facilitating the achievement of strategic goals.	I believe there is alignment between the strategic objectives outlined by my hospital's leadership and the day-to-day activities of employees. I believe the goals and priorities set at the organizational level communicated to different departments and teams, ensuring strategic alignment throughout the hospital. In my opinion the current communication strategy foster alignment with the overall strategic direction of the hospital.	[7] [8] [9]
		I perceive that individual performance goals align with the broader strategic objectives of the hospital.	[10]
		The connection between the mission and vision of the hospital and the specific strategic initiatives implemented to achieve them.	[11]
		In my hospital ensure that all levels of management and staff are aware of and committed to the strategic priorities set by the leadership team.	[12]
		The strategic plan reviewed and updated to ensure ongoing relevance and alignment with the dynamic healthcare landscape.	[12]
		I believe that the connection between the strategic objectives outlined in the Balanced Scorecard and the day-to-day activities and performance goals of employees at different levels.	[7]
Optimal Performance	Optimal performance refers to the highest level of efficiency, effectiveness, and productivity that an organization or individual can achieve. It involves maximizing output, achieving goals, and utilizing resources in the most efficient and effective manner to attain peak performance and desired outcomes.	I agree that certain key performance indicators (KPIs) are crucial for assessing the overall optimal performance of my hospital. I think the concept of optimal performance is communicated and understood among different levels of employees in your hospital. I believe that individual and team goals contribute to the achievement of optimal performance for the hospital. I believe that the hospital's current strategies for identifying and addressing areas for improvement to enhance overall performance of the hospital.	[8] [13] [8] [14]
		The current performance measurement and evaluation processes align with the hospital's goals for achieving optimal performance.	[9]
		I believe that the hospital's strategies or initiatives effectively foster a culture of continuous improvement to strive for optimal performance.	[15]
		You agree that leadership contributes to creating an environment that supports and encourages optimal performance among employees?	[12]
		I believe that the performance assessments conducted to ensure ongoing progress and alignment with the hospital's goals for optimal performance?	[12]

**Table 3:** Mean, standard deviation, reliability, skewness, and kurtosis of item.

Item	N	Mean	S.D	Reliability	Skewness	Kurtosis
BS1	530	2.991	1.2831	.798	-.117	-.959
BS2	530	2.811	1.2398	.801	.201	-.798
BS3	530	3.223	1.2341	.800	-.273	-.848
BS4	530	3.238	1.3174	.799	-.226	-1.033
BS5	530	2.309	1.2246	.793	.639	-.556
BS6	530	2.413	1.2843	.791	.489	-.856
BS7	530	2.289	1.2652	.792	.614	-.639
BS8	530	2.475	1.2050	.795	.565	-.435
SA1	530	2.191	1.1583	.790	.715	-.269
SA2	530	2.445	1.1576	.791	.431	-.430
SA3	530	2.583	1.2426	.797	.474	-.631
SA4	530	2.619	1.1658	.797	.368	-.493
SA5	530	2.466	1.1370	.792	.533	-.244
SA6	530	2.791	1.2464	.799	.390	-.734
SA7	530	2.675	1.1893	.792	.404	-.495
SA8	530	2.864	1.3218	.799	.187	-.998
OP1	530	2.711	1.2977	.802	.277	-.920
OP2	530	2.860	1.3027	.803	.157	-.998
OP3	530	2.811	1.2994	.800	.281	-.920
OP4	530	2.247	1.1492	.793	.721	-.204
OP5	530	2.296	1.1783	.791	.529	-.655
OP6	530	2.153	1.1912	.790	.706	-.422
OP7	530	2.455	1.1775	.796	.566	-.358
OP8	530	2.196	1.1450	.790	.740	-.177

**Fig. 6:**
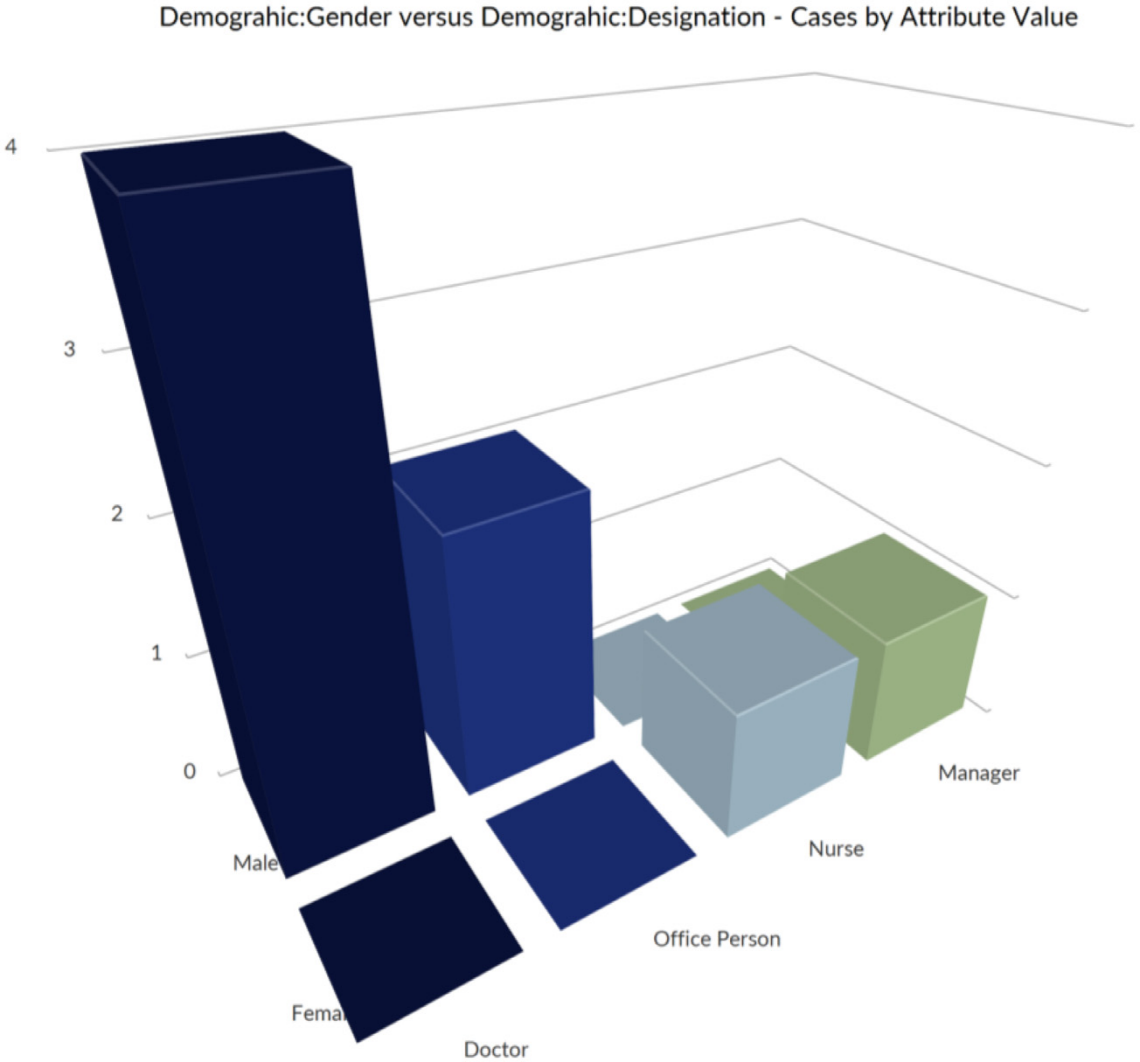
Distribution of cases by gender and designation.

**Fig. 7:**
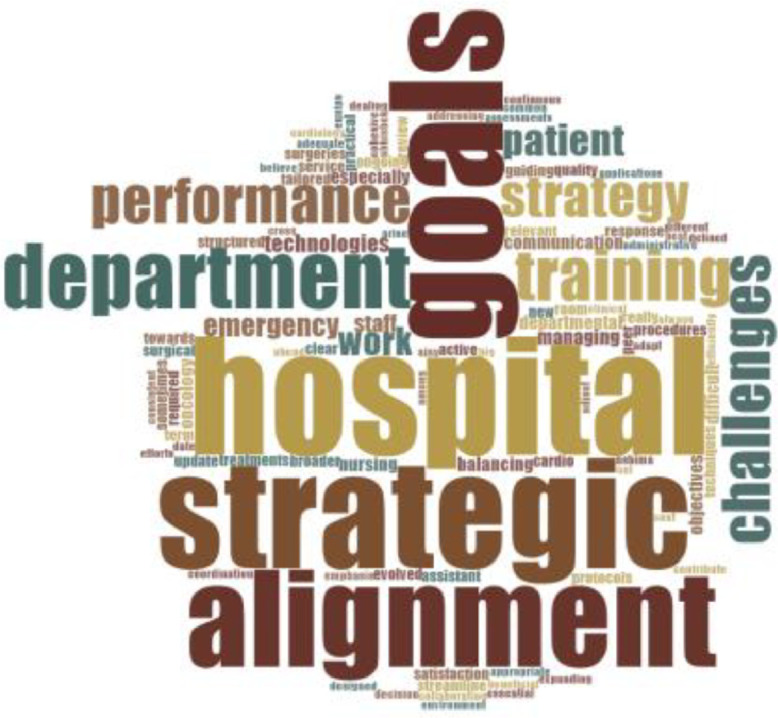
Word cloud.

**Fig. 8:**
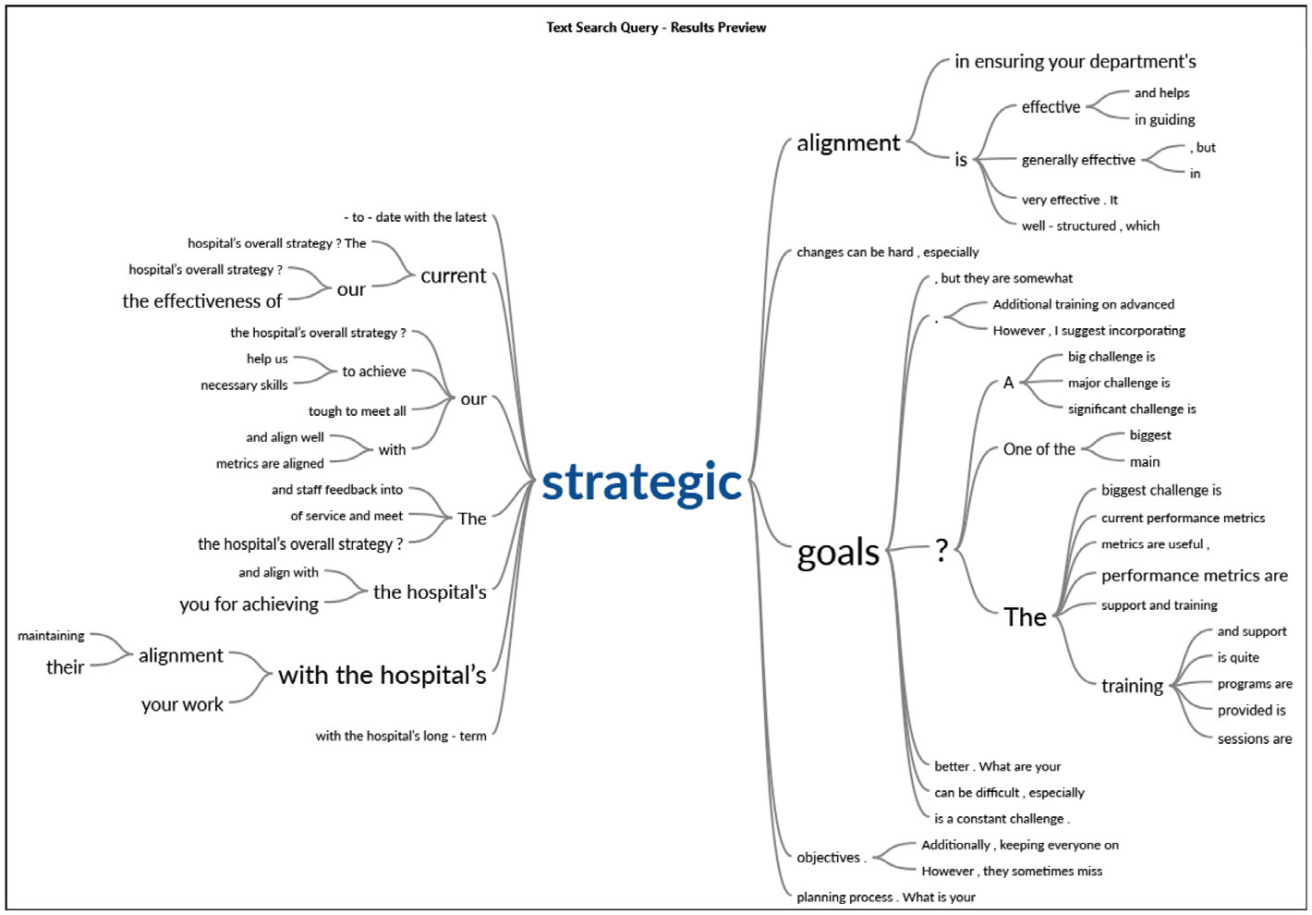
Word-tree.

We utilized NVivo 14 and IBM SPSS Statistics (Version 26) for data analysis. NVivo played a crucial role in analyzing qualitative data from interviews, allowing us to effectively code the responses. Fig. 6 displays the distribution of cases based on gender (Male and Female) across different designations (Doctor, Nurse, Office Person, Manager). The 3D bar chart visualizes the number of matching cases for each gender-designation combination, illustrating the gender representation within each professional role. Word clouds are shown in Fig. 7. Table 4 displays the sentiment distribution across the dataset, categorizing responses into very negative, moderately negative, moderately positive, and very positive groups. Fig. 8 represents the Word-Tree. This categorization helped us identify overall sentiment trends in the data. To maintain consistency and reliability, we used stop words to filter out frequently occurring but insignificant terms, ensuring our analysis focused on meaningful content.

**Table 4:** Sentiment distribution across the dataset.

Participant	Very negative	Moderately negative	Moderately positive	Very positive
Participant A	0	1	2	1
Participant B	0	0	3	1
Participant C	0	1	2	3
Participant D	0	1	1	2
Participant E	0	1	1	0
Participant F	1	0	3	0
Participant G	1	4	2	0
Participant H	0	1	3	1

For the quantitative analysis, SPSS was employed to explore relationships between the Balanced Scorecard, strategic alignment, and optimal performance. Table 5 presents the correlation matrix, highlighting the strength and direction of these relationships. Table 6 includes the ANOVA results, which reveal whether significant differences exist between group means. Lastly, Table 7 outlines the results of the regression analysis, demonstrating how well the Balanced Scorecard and strategic alignment predict performance outcomes.

**Table 5:** Correlation matrix of balanced scorecard, strategic alignment, and optimal performance.

		Balanced Scorecard	Strategic Alignment	Optimal Performance
Balanced Scorecard	Pearson Correlation	1	.300^⁎⁎^	.389^⁎⁎^
	Sig. (2-tailed)		.000	.000
	N	530	530	530
Strategic Alignment	Pearson Correlation	.300^⁎⁎^	1	.370^⁎⁎^
	Sig. (2-tailed)	.000		.000
	N	530	530	530
Optimal Performance	Pearson Correlation	.389^⁎⁎^	.370^⁎⁎^	1
	Sig. (2-tailed)	.000	.000	
	N	530	530	530

**Table 6:** ANNOVA table.

Model	Sum of Squares	df	Mean Square	F
Regression	3306.267	2	1653.134	75.116
Residual	11598.150	527	22.008	
Total	14904.417	529

**Table 7:** Regression table.

Model	Unstandardized Coefficients		Standardized Coefficients	t	Sig.
	B	Std. Error	Beta		
Constant	8.066	.973		8.287	**.000**
Strategic Alignment	.260	.038	.279	6.917	**.000**
Balance Scorecard	.290	.038	.305	7.580	**.000**

## Limitations

The study's findings suggest a significant positive relationship between Strategic Alignment, Balanced Scorecard (BSC) implementation, and Optimal Performance (OP) in multispecialty hospitals. However, the study's cross-sectional design, reliance on self-reported data, and limited generalizability to other healthcare settings and regions are notable limitations. Future research should consider longitudinal or experimental designs, objective performance measures, and diverse healthcare contexts to enhance the validity and generalizability of the findings.

## Ethics Statement

The research extensively utilized nonexperimental voluntary surveys as the primary method of data collection. It is noteworthy that the research was carried out in an environment where formal ethical approval was not mandatory for survey-based studies. This decision was made after careful consideration of the nature of the research and the prevailing guidelines within the research environment. In accordance with ethical principles, the privacy of participants was a paramount concern throughout the study. Notably, no personally identifiable information was solicited from the participants during the survey administration. This deliberate measure was taken to ensure the confidentiality and anonymity of the participants. The research team took proactive steps to secure the voluntary participation of individuals, and participants were fully informed about the purpose and objectives of the study. Their participation was entirely voluntary, and they were made aware of their right to withdraw from the study at any point without facing any consequences.

## CRediT authorship contribution statement

**Shefali Mohan:** Writing – original draft, Conceptualization, Formal analysis, Methodology. **Rohit Kushwaha:** Conceptualization, Methodology, Writing – review & editing, Formal analysis. **M. Venkatesan:** Formal analysis, Conceptualization. **Ramya Singh:** Conceptualization, Methodology, Writing – review & editing. **Manish Mishra:** Data curation, Formal analysis.

## Data Availability

Mendeley DataSurvey data on Strategic alignment in multispecialty hospitals: Implementing a balanced scorecard approach for optimal performance (Original data).

## Appendix Supplementary materials

Image, application 1
