# Supplementary material for: Survey data on strategic alignment in multispecialty hospitals: Implementing a balanced scorecard approach for optimal performance
Source: Data Brief. 2025 Jan 31;59:111329. doi: 10.1016/j.dib.2025.111329 (PMC11849655; doi:10.1016/j.dib.2025.111329)
Supplement: Supplementary file 1 [file mmc1.docx]

**Interviewee 1: Dr Col Ajay - Cardio, Apollo Hospital, Lucknow**

1. **How do you perceive the effectiveness of our current strategic alignment in ensuring your department's goals are in line with the hospital’s overall strategy?**
   Our department's goals are well-aligned with the hospital's overall strategy, which has really helped us streamline our processes and improve patient care. Also I think involving all departmental heads in the strategy-making process could make this alignment even more effective.
2. **What is your opinion on the training and support provided to you for achieving the hospital's strategic goals?**
   The training sessions are thorough and cover a wide range of essential topics and understandings, which is great for achieving our goals. However, it would be even better if the training were structured/ tailored to the specific needs of each department.
3. **What are your thoughts on the current performance metrics used to evaluate your department’s success and their alignment with the hospital’s strategic goals?**
   The performance metrics are clearly defined and align well with our strategic objectives. However, they sometimes miss the qualitative aspects of patient care, which are really important in a department like cardiology.
4. **Can you describe the biggest challenges you face in aligning your work with the hospital’s strategic goals?**
   A big challenge is managing the high patient volume, which can stretch our resources and make it tough to meet all our strategic objectives. Additionally, keeping everyone on the team up-to-date with the latest strategic changes can be hard, especially given the fast pace of our work.

**Interviewee 2: Dr Tarun - Cardio, Apollo Hospital, Mumbai**

1. **How do you perceive the effectiveness of our current strategic alignment in ensuring your department's goals are in line with the hospital’s overall strategy?**
   The current strategic alignment is very effective. It helps ensure that our surgical procedures are not only successful but also contribute positively to the hospital's reputation and financial goals.
2. **What is your opinion on the training and support provided to you for achieving the hospital's strategic goals?**
   The support and training provided are excellent, focusing on the latest surgical techniques and patient care strategies. More frequent refreshers would be helpful to keep up with the rapid advancements in our field.
3. **What are your thoughts on the current performance metrics used to evaluate your department’s success and their alignment with the hospital’s strategic goals?**
   The performance metrics are fairly robust ,active and provide a good measure of our success. However, incorporating more intense feedback from patients post-surgery could provide a more comprehensive view of our performance.
4. **Can you describe the biggest challenges you face in aligning your work with the hospital’s strategic goals?**
   The biggest challenge is managing the expectations of both the hospital management and the patients. Balancing cost-effective surgeries with high-quality patient care is an ongoing, continuous challenge.

**Interviewee 3: Dr Yogesh - Physiotherapist Apollo Hospital, Lucknow**

1. **How do you perceive the effectiveness of our current strategic alignment in ensuring your department's goals are in line with the hospital’s overall strategy?**
   Our strategic alignment is generally effective, but there's a gap in communication between different levels of staff, which sometimes leads to misunderstandings about the hospital's goals.
2. **What is your opinion on the training and support provided to you for achieving the hospital's strategic goals?**
   The training is quite thorough, but there needs to be more emphasis on practical applications rather than just theoretical knowledge, especially for nursing staff who are on the frontline.
3. **What are your thoughts on the current performance metrics used to evaluate your department’s success and their alignment with the hospital’s strategic goals?**
   The metrics are useful, but they could be improved by including more qualitative measures, such as patient satisfaction and staff morale.
4. **Can you describe the biggest challenges you face in aligning your work with the hospital’s strategic goals?**
   One of the main challenges is the high turnover rate among nursing staff, which makes it difficult to maintain a consistent level of service and meet the strategic goals.

**Interviewee 4: Gopal - Office Assistant, Apollo Hospital, Hyderabad**

1. **How do you perceive the effectiveness of our current strategic alignment in ensuring your department's goals are in line with the hospital’s overall strategy?**
   The strategic alignment is effective in guiding our department's activities towards the hospital's broader goals. However, there's always room for improvement in terms of integrating patient and staff feedback into the strategic planning process.
2. **What is your opinion on the training and support provided to you for achieving the hospital's strategic goals?**
   The training provided is adequate but could be more paediatric-specific. Tailoring the training to our department's unique and specific needs would help us to achieve our strategic goals better.
3. **What are your thoughts on the current performance metrics used to evaluate your department’s success and their alignment with the hospital’s strategic goals?**
   The performance metrics are aligned with our goals, but they are heavily focused on quantitative data. Incorporating more qualitative metrics would provide an evolved picture of our department's performance.
4. **Can you describe the biggest challenges you face in aligning your work with the hospital’s strategic goals?**
   A significant challenge is the rapidly changing healthcare environment, which requires us to constantly adapt our strategies. Keeping up with these changes while maintaining alignment with the hospital's goals is challenging.

**Interviewee 5: Ms. Reena, Nurse, Apolo Hospital, Lucknow**

1. **How do you perceive the effectiveness of our current strategic alignment in ensuring your department's goals are in line with the hospital’s overall strategy?**
   The strategic alignment is generally effective in aligning our department's objectives with the hospital's broader goals. However, there is a need for more frequent updates and feedback sessions to ensure that everyone is on the same page and that our strategies remain relevant.
2. **What is your opinion on the training and support provided to you for achieving the hospital's strategic goals?**
   The training and support are comprehensive, covering both clinical and administrative aspects. However, additional training on emerging technologies and treatments in oncology would help us stay ahead in our field.
3. **What are your thoughts on the current performance metrics used to evaluate your department’s success and their alignment with the hospital’s strategic goals?**
   The performance metrics are effective, but they tend to focus more on the quantitative aspects of our performance. Including patient satisfaction and quality of care measures would provide a more holistic view of our department's success.
4. **Can you describe the biggest challenges you face in aligning your work with the hospital’s strategic goals?**
   One of the main challenges is the rapidly evolving nature of oncology treatments and technologies. Keeping our strategies aligned with the hospital’s goals while incorporating the latest advancements in oncology can be difficult.

**Interviewee 6: Dr Abhishek - Emergency manager, Apolo Hospital**

1. **How do you perceive the effectiveness of our current strategic alignment in ensuring your department's goals are in line with the hospital’s overall strategy?**
   The strategic alignment is well-structured, which helps us in setting clear departmental goals that support the hospital's overall strategy. However, there is room for improvement in cross-departmental communication to ensure more cohesive efforts.
2. **What is your opinion on the training and support provided to you for achieving the hospital's strategic goals?**
   The training programs are well-designed and relevant. However, I believe there should be more opportunities for peer learning and sharing best practices across departments.
3. **What are your thoughts on the current performance metrics used to evaluate your department’s success and their alignment with the hospital’s strategic goals?**
   The performance metrics are aligned with our strategic goals, but they are somewhat limited in scope. Expanding these metrics to include community outreach and preventive health measures would provide a more comprehensive view of our impact.
4. **Can you describe the biggest challenges you face in aligning your work with the hospital’s strategic goals?**
   The biggest challenge is the required coordination across various departments to implement new strategies effectively. Ensuring that all departments work towards common goals while maintaining their individual responsibilities can be challenging.

**Interviewee 7: Ms Ashima - HR Apollo Hospital**

1. **How do you perceive the effectiveness of our current strategic alignment in ensuring your department's goals are in line with the hospital’s overall strategy?**
   Our current strategic alignment is effective in guiding our emergency response protocols in line with the hospital's mission and vision. Nonetheless, real-time feedback mechanisms could enhance this alignment by addressing issues promptly as they arise.
2. **What is your opinion on the training and support provided to you for achieving the hospital's strategic goals?**
   The training provided is thorough and equips us to handle emergencies efficiently. However, ongoing training sessions to update us on the latest emergency protocols and technology would be beneficial.
3. **What are your thoughts on the current performance metrics used to evaluate your department’s success and their alignment with the hospital’s strategic goals?**
   The current performance metrics are appropriate and align with the hospital's strategic goals. However, I suggest incorporating more patient outcome-based metrics to gauge the effectiveness of our emergency services with increased vigilence
4. **Can you describe the biggest challenges you face in aligning your work with the hospital’s strategic goals?**
   A major challenge is dealing with the unpredictability of emergencies. Balancing the need for quick decision-making with the hospital’s long-term strategic goals can be difficult, especially in high-pressure situations.

**Interviewee 8: Mr Varun - Assistant, Apolo Hospital, Lucknow**

1. **How do you perceive the effectiveness of our current strategic alignment in ensuring your department's goals are in line with the hospital’s overall strategy?**
   The strategic alignment is effective and helps streamline our processes. Regular meetings to review and adjust our strategies based on the hospital’s overall goals would further enhance this alignment.
2. **What is your opinion on the training and support provided to you for achieving the hospital's strategic goals?**
   The training sessions are excellent and provide us with the necessary skills to achieve our strategic goals. Additional training on advanced imaging techniques and interdepartmental collaboration would be valuable.
3. **What are your thoughts on the current performance metrics used to evaluate your department’s success and their alignment with the hospital’s strategic goals?**
   The performance metrics are aligned with our goals but focus too much on quantitative data. Including more qualitative assessments, such as peer reviews and patient feedback, would provide a more balanced and wholistic view of our department’s performance.
4. **Can you describe the biggest challenges you face in aligning your work with the hospital’s strategic goals?**
   One of the biggest challenges is keeping up with the rapid technological advancements in radiology. Integrating new technologies and procedures while maintaining alignment with the hospital’s strategic goals is a constant challenge.
